# Development of an in vitro three dimensional loading-measurement system for long bone fixation under multiple loading conditions: a technical description

**DOI:** 10.1186/1749-799X-2-21

**Published:** 2007-11-24

**Authors:** John C Janicek, William L Carson, David A Wilson

**Affiliations:** 1 From the University of Missouri Comparative Orthopaedic Laboratory, 379 East Campus Drive, Columbia, MO, 65211, USA

## Abstract

The purpose of this investigation was to design and verify the capabilities of an in vitro loading-measurement system that mimics in vivo unconstrained three dimensional (3D) relative motion between long bone ends, applies uniform load components over the entire length of a test specimen, and measures 3D relative motion between test segment ends to directly determine test segment construct stiffness free of errors due to potting-fixture-test machine finite stiffness.

Intact equine cadaveric radius bones, which were subsequently osteotomized/ostectomized and instrumented with bone plates were subjected to non-destructive axial, torsion, and 4-point bending loads through fixtures designed to allow unconstrained components of non-load associated 3D relative motion between radius ends. 3D relative motion between ends of a 50 mm long test segment was measured by an infrared optical tracking system to directly determine its stiffness. Each specimen was then loaded to ultimate failure in either torsion or bending. Cortical bone cross-section diameters and published bone biomechanical properties were substituted into classical mechanics equations to predict the intact test segment theoretical stiffness for comparison and thus loading-measurement system verification.

Intact measured stiffness values were the same order of magnitude as theoretically predicted. The primary component of relative motion between ends of the test segment corresponded to that of the applied load with the other 3D components being evident and consistent in relative magnitude and direction for unconstrained loading of an unsymmetrical double plate oblique fracture configuration. Bone failure configurations were reproducible and consistent with theoretically predicted.

The 3D loading-measurement system designed: a) mimics unconstrained relative 3D motion between radius ends that occurs in clinical situations, b) applies uniform compression, torsion, and 4-point bending loads over the entire length of the test specimen, c) measures interfragmentary 3D relative motion between test segment ends to directly determine stiffness thus being void of potting-fixture-test machine stiffness error, and d) has the resolution to detect differences in the 3D motion and stiffness of intact as well osteotomized-instrumented and ostectomized-instrumented equine radii.

## Background

Biomechanical studies that appear in the literature typically use fixtures that constrain some components of relative 3D motion between ends of the test specimen, other than in the direction of loading, and/or do not apply a uniform component of load over the instrumented length of the specimen [1-8], and use test machine ram displacement to determine stiffness without correcting for potting-fixture-test machine (PFT) stiffness error [1,2,4,6,7,9]. Direct measurement of relative 3D interfragmentary motions during in vitro testing has been performed [5,7,10-12], including those that have eliminated PFT stiffness [11,12]. In vivo 3D interfragmentary motions have been measured using a transducer telemetry system [13], a motion capture system [6,7,9], and a 3D optical tracking system [14]. To the authors' knowledge, combined use of 3D unconstrained fixtures with 3D optical tracking of test segment ends to eliminate PFT stiffness error has not appeared in human or veterinary literature.

The purpose of the investigation described in this paper was to design and verify the capabilities of an in vitro loading-measurement system that: 1) mimics in vivo unconstrained relative 3D motion between fracture-model (herein after referred to as "fracture") segments of long bone ends, 2) applies uniform compression, torsion, or bending moment loads over the entire length of the test specimen, 3) measures 3D relative motion between test segment ends to directly determine 3D stiffness components of intact and instrumented test segments, and 4) identifies the weakest aspects of an instrumented specimen during increased uniform loading over its entire length. The ultimate goal was to use this loading-measurement system to measure and compare the relative 3D biomechanical characteristics of intact and two Association for the Study of Internal Fixation (ASIF) techniques for repair of an oblique fracture involving the distal (inferior) diaphysis of an equine radius.

## Methods

### Loading fixture design, and potting

Proximal (superior) and distal steel box type loading fixtures were designed to compliment application of ASIF devices along the distal aspect of the lateral and cranial (anterior) radius surfaces with at least a 0.5 cm distance from potting material, and to have sufficient bone-potting interface to transmit loads to failure of the instrumented test segment.

**Figure 1:**
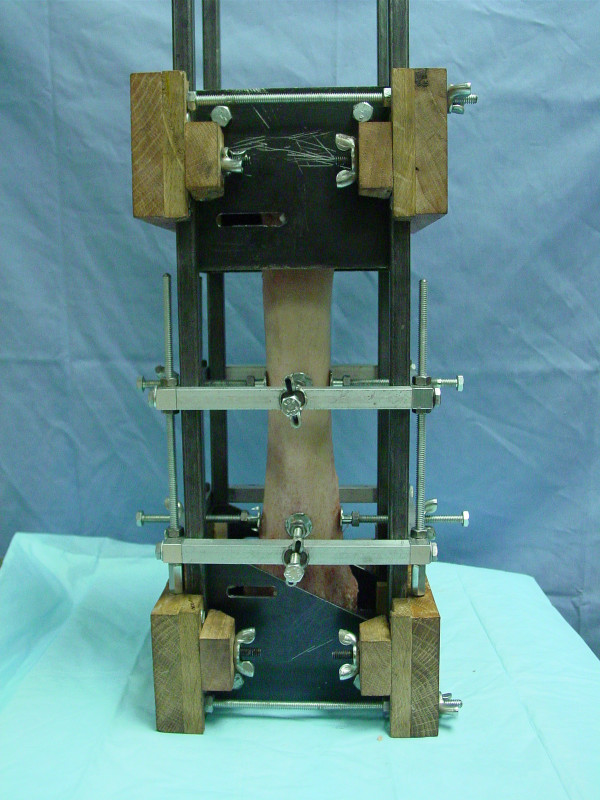
Steel box-type loading fixtures placed within potting jig, CrCa view of radius.

A potting jig was designed to align and hold the proximal and distal loading fixtures and the radius during potting (Figure 1). The long axis of the radius was aligned by locating the major and minor diameter periosteal surfaces equidistance from the sides of the fixtures at two cross sections, one 10 cm from the distal end of the radius and the other being 10 cm proximal to it. This placed the long axis of the radius coincident with the center of the spherical socket centrally located in the proximal and distal loading fixtures. To help secure the radius to the fixtures during application of bending moments, a cranial-to-caudal (CrCa) directed steel stud was passed through slots in the loading fixture sides and a hole drilled through the medial side of both ends of the radius. The ends of the radius were potted by using a mixture by volume of 70% limestone (screened 0.32 to 0.64 cm) and 30% dry polymethylmethacrylate (PMMA) powder (Technovit, Jorgenson Laboratories, Loveland, CO) wetted with methacrylate monomer to make pourable slurry.

**Figure 2:**
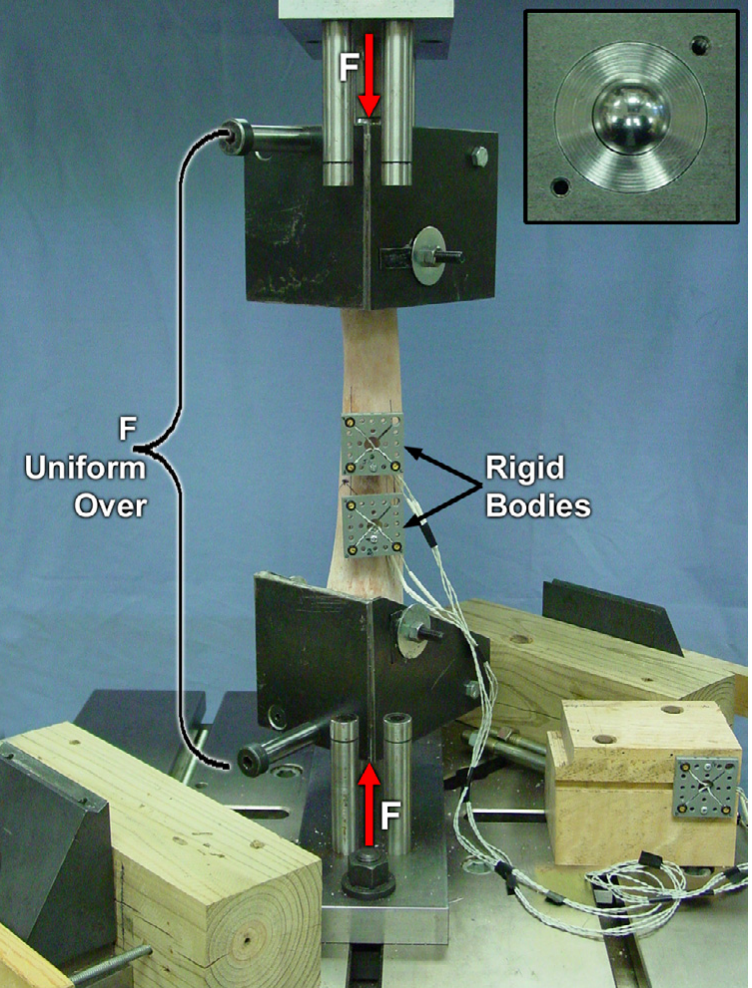
Axial compression (F) applied through hardened steel spheres. This allowed unconstrained 3D relative movement of the test specimen ends, and produced uniform load over the entire length of the specimen. Insert: view of hardened steel sphere in each loading fixture base during axial compression.

Using potting jig uprights as a reference, center lines were drawn on four orthogonal surfaces of the radius. The proposed fracture plane was marked from a point 4 cm proximal to the lateral styloid process to a point 12 cm proximal to the medial styloid process. Cross section ends of the 50 mm long test segment were located and marked at the level of the fracture plane's center and 50 mm proximal to it. A hole was drilled and tapped (#8–32 thread) into the dorsomedial cortex of right radii and the palmaromedial cortex for left radii to securely mount a 3D optical measuring system rigid body to each end of the test segment (Figure 2).

### Unconstrained 3D loading system design

An Instron 8821S (Instron, 100 Royall Street, Canton, MA) biaxial loading frame equipped with a 25 kN axial, ± 225 Nm torque load cell was used along with the loading fixtures described below to apply a 3D component of load uniformly over the length of the radius. Position control was used to move the global component at a constant rate of relative displacement between radius ends that corresponded to the applied load component. Assuming negligible friction in the lubricated joints, the fixtures were designed to produce no external constraints on the remaining (out of 6 total) 3D components of proximal relative to distal end displacement of the radius (herein after called "unconstrained"). The weight of the radius and fixtures were assumed to be negligible compared to the applied loads.

#### Axial compression

Axial compression was applied through hardened 25.4 mm diameter steel spheres centrally located in the end of each loading fixture base (Figure 2). Because of freedom of rotation of both ends about the sphere joints, there were no external constraints on proximal relative to distal end of radius: lateral-to-medial (LM) and CrCa translation (shear); and axial, LM and CrCa bending rotations.

#### Torsion

**Figure 3:**
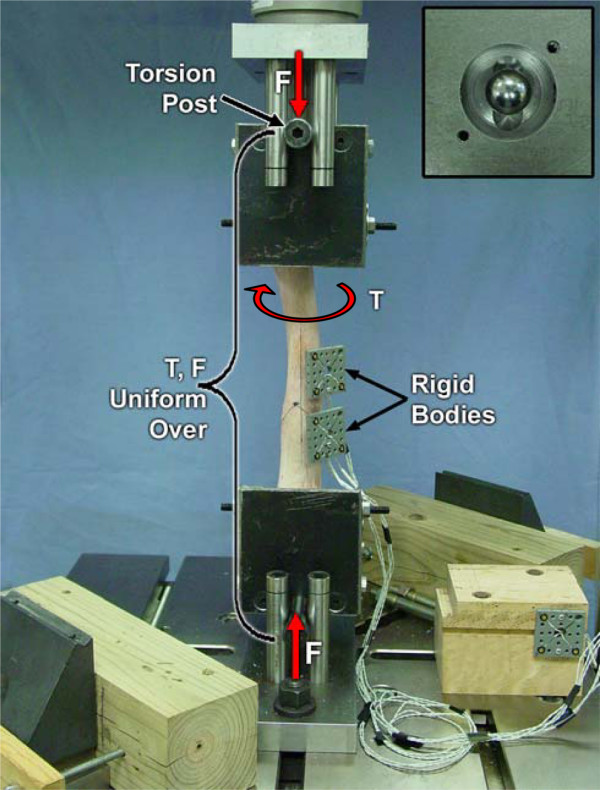
Torsion (T) applied by transmitting equal but opposite components of force to hardened steel shoulder bolts on the lateral and medial side of each fixture, with the option to apply an axial bias load (F). This allowed unconstrained 3D relative movement of the test specimen ends, and produced uniform load over the entire length of the specimen. Insert: view of hardened steel sphere free to move in a CrCa spherical slot in each loading fixture base keeping the fixture centered LM while allowing all CrCa components of force to be transmitted through the pair of shoulder bolts.

Torsion was applied by transmitting equal but opposite direction components of tangential force to hardened steel shoulder bolts attached to the lateral and medial sides of each fixture on a common axis through the center of the centrally located sphere (Figure 3). An axial bias load could simultaneously be applied through the hardened steel sphere, which was free to move in a CrCa spherical slot in each fixture base. Because of freedom of LM and CrCa rotation of both ends about the sphere joints, there were no external constraints on proximal relative to distal end of radius: LM and CrCa translation; and LM and CrCa bending rotations.

#### 4-point bending

**Figure 4:**
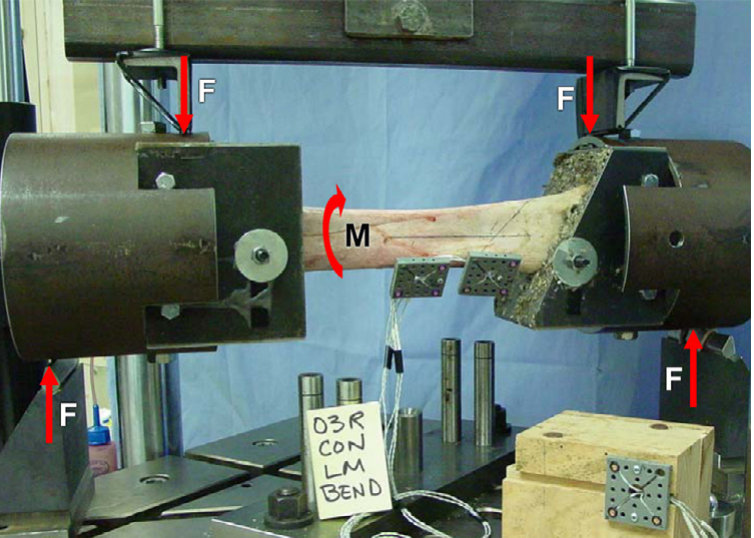
4-point LM bend. Sections of 15.2 cm diameter steel pipe are bolted to each fixture base to which 4-point bending loads (F) are applied without contacting the bone or bone implants.

**Figure 5:**
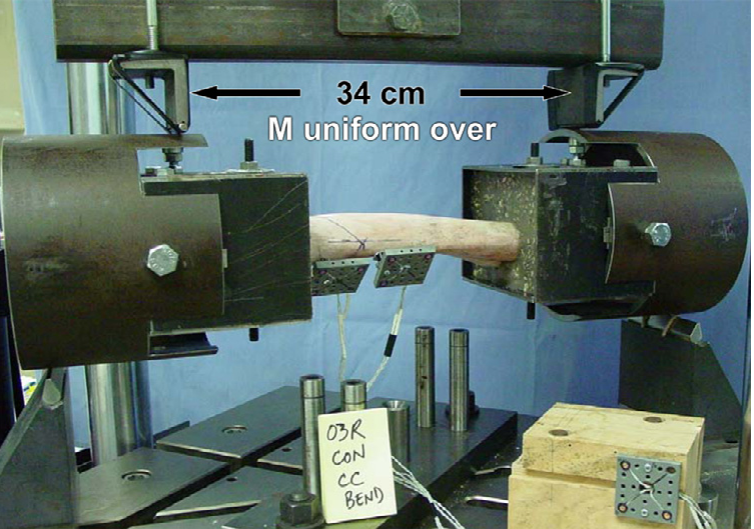
4-point CrCa bend. Test specimen rotated 90 degrees about its longitudinal axis in the 4-point loading apparatus to produce CrCa bend. Four-point bending loads are applied 10 cm apart, leaving the length of the exposed radius plus fixtures (34 cm) subject to a uniform bending moment (M).

Sections of 15.2 cm diameter (0.64 cm wall thickness) steel pipe were bolted with shims using the shoulder bolt holes to each fixture base (Figure 4), and stiffened with miniature screw type jacks placed in the plane of loading. Four-point bending forces were applied on the pipe 10 cm apart through transversely oriented 0.64 cm diameter hardened steel surfaces. The inner load application surfaces were attached to an arm equidistance from its pivot, thus creating equal inner forces regardless of how or where the test radius deformed and/or failed. This created a 34 cm length of radius plus potted ends subject to a uniform bending moment (Figure 5). The pipe allowed the specimen-fixture assembly to be rotated 90 degrees about its longitudinal axis to load the radius in LM, CrCa, medial-to-lateral (ML), and caudal-to-cranial (CaCr) bending. Assuming no friction between pipe and transverse loading surfaces, there were no external constraints on proximal relative to distal end of radius: axial, LM and CrCa translation; axial rotation, and bending rotation transverse to the plane of loading.

### 3D relative inter-fragmentary motion measurement system

3D motion of the ends of the 50 mm long test segment were measured as load was applied by using a 0.01 mm resolution 3D infrared close focus Certus optical tracking system (NDI Optotrak, Waterloo, Ontario, Canada) to follow the light emitting diodes (LEDs) in the PVC rigid bodies attached at each end of the test segment using a #8–32 machine screw (Figure 2). A third rigid body was attached to a reference block on the base of the testing machine. The reference block had three miniature conical indentations located at known distances from the test machine's Z axis: one at the origin and one each on mutually perpendicular global X and Y axes. A 0.1 mm diameter tip portable digitizing probe was used to digitize the location of the three indentations on the reference block to define a global XYZ coordinate system, and 5 predetermined points around the fracture plane: one on each side of the lateral plate, one adjacent to cranial plate, and one each on the medial and caudal surface centerlines. Point digitization was done after each specimen was mounted in torsion fixtures with the medial side in the X direction. The digitized points and the optical tracking system's software was then used to define a local xyz_d _orthogonal coordinate axes associated with the rigid body attached at the distal end of the 50 mm long test segment, z_d _coincident with the radius' axis and x_d _in the medial direction. Similarly, a local xyz_p _coordinate axes was defined and associated with the rigid body attached at the proximal end. The local axes remained oriented with respect to and attached to the distal and proximal rigid bodies, thus tracking the 3D position of the radius' cross section at each end of the test segment as the specimen was placed in and loaded in each mode of loading.

### Test segment stiffness determination and test system verification

To verify the capabilities of the 3D loading-measurement system, the stiffness associated with each mode of loading was measured for intact radius test segments (n = 20), and compared to the corresponding stiffness theoretically predicted with equations of classical mechanics. Each radius was then osteotomized, instrumented with ASIF techniques, and subjected to the same sequence of randomized 3-cycle non-destructive load tests (n = 20). Eight osteotomies were then converted to 7 mm ostectomies, re-instrumented, and subjected to the same sequence of 3-cycle non-destructive load tests. Representative results of the intact, osteotomized, and ostectomized tests for each load mode were compared to each other to determine: if the measurement system could resolve the primary component of 3D motion corresponding to direction of applied load from the other components, and if it had sufficient resolution to measure the stiffness of intact segments for comparison to that of fractured segments secured with different types of internal fixation. Following the last of these tests, each radius was subjected to either a 1-cycle torsion, LM bend or CrCa bend to failure test. The location and orientation of the fracture surfaces were observed and compared to the classical brittle fractures that would be produced by a pure component of applied load to help verify that the loading system is indeed applying the desired "pure" component of load.

#### Measured stiffness

Each intact radius was subjected to a 3-cycle non-destructive random sequence of load modes: axial compression, torsion, and CrCa and LM bending (n = 12), and in addition CaCr and ML bending (n = 8). Instron's RSBasLab software was used to apply load at a constant ram displacement rate (0.004 mm/sec axial compression, 0.25 deg/sec torsion, and 0.02 mm/sec bending), with load limits used to define ends of a cycle (100 to 4000 N axial compression, ± 50 Nm torsion, and 10 to 150 Nm bending). The 3D position of the rigid body LEDs were synchronously measured and stored along with the testing machine's ram position and applied load at sampling rates of 50 Hz axial, and 100 Hz torsion and bending. NDI Toolbench software was used to determine the 3D position of the proximal end of the test segment relative to the distal end for each test and to transfer this data along with applied load into an EXCEL file. EXCEL was used to plot the 3D components of relative position as a function of the component of applied load.

The component of relative displacement between test segment ends was plotted as a function of the corresponding applied load component. Measured stiffness was determined as the reciprocal of the slope of a straight line fit to this data between selected load ranges, typically having linear data, during the 3rd loading cycle: 200 to 4000 N for axial compression, 10 to 50 Nm for positive torque, -10 to -50 Nm for negative torque, 10 to 150 Nm for bending.

To compare the intact measured radius stiffness results (herein after called K_3D_) to those published by Hanson et al. [1] who used ram displacement in determining stiffness values (herein after called K_H_), the following was performed. Hanson et al's [1] axial and torsion tests were conducted on a 150 mm long test segment, thus the K_3D _results were multiplied by the ratio of test segment lengths (50 mm/150 mm) to obtain corresponding 150 mm segment length normalized values (herein after called K_3DN_) for direct comparison K_H _results. Hanson et al's [1] 4-point bending tests were conducted with an apparatus having 11.5 mm between inner force application points and 101 mm between outer force application points, with both inner and outer forces applied directly to bone. Hanson et al. [1] did not report how relative angular displacement between cross sections was determined from the ram's axial displacement or between which pair of cross sections. Thus we converted our measured K_3D _values to those for a uniform bending segment of 11.5 mm and 101 mm by multiplying by the ratio of test segment lengths (50 mm/11.5 mm) = 4.35 and (50 mm/101 mm) = 0.50 respectively, to determined a range of K_3DN _values within which K_H _values should theoretically lie.

#### Theoretical segment stiffness

After testing to failure, each radius was transected 1 cm distal to the proximal rigid body allowing periosteal and endosteal cross section major and minor diameters to be measured with digital calipers. Two cross section shapes, hollow ellipse and hollow rectangle, were assumed to calculate a range of theoretically predicted stiffness values that would account for the non-uniform shape of the radius. The measured cross section diameters and published bone biomechanical properties were substituted into classical mechanics stiffness equations to predict its compression, torsion and bending stiffness values (Appendix).

## Results

**Figure 6:**
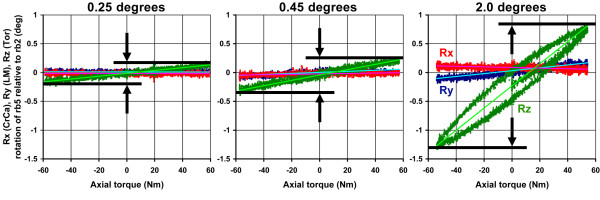
Example of the 3D components of rotational displacement between ends of the 50 mm test segment versus applied torque (-50 to +50 Nm): A) intact control, B) osteotomy, and C) ostectomy. Note that the system resolution was capable of distinguishing total axial rotation of only 0.25, 0.45, and 2.0 degrees, respectively for these three models with notably less CrCa and LM bending rotation.

Major periosteal and endosteal cross section diameters measured 49.8 (± 4.4) mm and 30.5 (± 4.8) mm, respectively, whereas the minor periosteal and endosteal cross section diameters measured 32.1 (± 1.4) mm and 18.4 (± 1.7) mm, respectively. The mean intact measured K_3D _values were within the average elliptical to rectangular cross section theoretical range for axial compression, torsion and ML bend; and were greater than the highest theoretically predicted by a factor of 1.1 for LM bend, 1.3 for CaCr bend, and 1.5 for CrCa bend (Table 1). The length normalized axial and torsion K_3DN _results were a factor of 19.6 and 7.5 higher, respectively, than the corresponding K_H _results (Table 2). Likewise, the 101 mm CrCa and LM bending K_3DN _results were a factor of 8.3 and 10.6 higher than the corresponding K_H_, respectively; whereas, the 11.5 mm CrCa and LM bending K_3DN _results were a factor of 72 and 92 higher (Table 2). The fixtures allowed components of 3D motion other than in the direction of loading to occur. For axial and torsional loading, unconstrained relative 3D motion across the fracture site was most noticeable for the ostectomy due to definite lack of load sharing with bone at the fracture site and the unsymmetrical double plate construct. The least amount of 3D motion between ends of the test segment occurred for the intact radius. This is illustrated in the torsion loading example which shows detectable CrCa and LM bending rotation for the ostectomy, and negligible CrCa and LM bending rotation for the intact radius and the reduced osteotomy (Figure 6). The loading fixtures during axial and torsion testing were also observed to have CrCa and LM bending rotation, which was most prevalent for the ostectomy model. Relative 3D motion between ends of the test segment was detected by the optical tracking system for bending loads which in some cases was visible by the relative motion across the fracture; however, 3D motion of the loading fixtures was not readily visible.

**Table 1:** 3D loading-measurement system verification. Measured intact equine radii stiffness values for selected load ranges of the third loading cycle were observed to be the same order of magnitude as theoretically predicted values for compression, torsion, and 4-point bending.

	**Mean (± SD) stiffness of 50 mm long test segment**						
	
	**Axial Compression (N/mm)**	**+ Torsion (Nm/deg)**	**- Torsion (Nm/deg)**	**CrCa Bend (Nm/deg)**	**CaCr Bend (Nm/deg)**	**LM Bend (Nm/deg)**	**ML Bend (Nm/deg)**
**Load Ranges**	200 to 4000 N	10 to 50 Nm	-10 to -50 Nm	10 to 150 Nm			
**Measured using: **3D loading-measurement system							
**Intact Control**	295000 (± 130977)	+430 (± 49)	-405 (± 57)	1007 (± 258)	885 (± 117)	1680 (± 395)	1452 (± 368)
**Theoretical using: **classical mechanics equations, cross-section measured dimensions, & two shapes							
**Ellipse (hollow)**	278000 (± 15261)	250 (± 26)		400 (± 49)		879 (± 94)	
**Rectangle (hollow)**	354000 (± 35277)	556 (± 57)		679 (± 83)		1490 (± 159)	

Bone properties assumed: Modulus of Elasticity (E) = 18000 N/mm^2^. Range in literature: (8900 ≤ E ≤ 42000)

Shear Modulus (G) = 4615 N/mm^2^. Range in literature: (3500 ≤ G ≤ 8710)

**Table 2:** Comparison of length normalized 3D loading-measurement system mean stiffness (K_3DN_) results to published conventional test machine load-measurement system stiffness (K_H_) results of intact equine radii. Differences in testing protocols are listed in the table. To be able to directly compare our 50 mm test segment stiffness (K_3D_) results, they were normalized to comparable values for test segment lengths used by Hanson et al. [1] by multiplying K_3D _by the ratio of test segment lengths: for axial and torsion (50 mm/150 mm), and for bending (50 mm/11.5 mm) and (50 mm/101 mm) to produce a range of K_3DN _values within which K_H _should lie.

	**Mean stiffness (K) of intact equine radius**			
	
	**Axial Compression (N/mm)**	**+/-Torsion (Nm/deg)**	**CrCa Bend (Nm/deg)**	**LM Bend (Nm/deg)**
**Measured using: **3D unconstrained fixtures **(3D optical) **34 cm test section 4-point bend, force applied on steel extensions to potted radius ends 3D optical tracking of the 50 mm test segment ends				
**K**_**3D **_(50 mm test segment)	295000	430/405	1,007	1,680
**K**_**3DN **_(150 mm axial & torsion segment) (11.5 to 101 mm uniform bend segment)	98000	143/135	4378 to 504	7305 to 840
**Measured using: **Constrained axial compression and torsion fixtures **(Hanson et al.) **1.15 cm test section 4-point bend (10.1 cm between outer force applicators), force applied directly on bone. Test machine ram displacement				
**K**_**H **_(150 mm axial & torsion segment) (11.5 mm between inner, 101 mm between outer 4-point bend force application points)	5000	18	61	79

Construct failure configurations were consistent with theoretical failure modes for brittle material (bone) and reproducible for both the torsion and bending load to failure tests. Torsion failure was consistently attributable to spiral fracture propagation that initiated perpendicular to the osteotomy or ostectomy at cranial screw holes. LM and CrCa bend failure initiated by cis-cortex fracturing through either the proximal or distal screw holes adjacent to the osteotomy or ostectomy, and progressed perpendicular to the longitudinal axis of the radius; with the exception of one ostectomy undergoing LM bend to failure experiencing substantial bone-plate interface separation due to lateral screw pull out of bone without any bone fracture. LM and CrCa bends produced visible osteotomy and ostectomy gap opening on the non-plated side due to it being in tension, and visible proximal and distal screw bending adjacent to the osteotomy or ostectomy. ML and CaCr bending produced visible ostectomy gap closure on the non-plated side due to it being in compression.

No failures occurred within the potted ends or of the fixtures themselves during torsional and bending loads to failure. No failures occurred through rigid body screw holes.

## Discussion

Measured stiffness (K_sm_) of a long bone specimen is a function of parameters associated with morphologic/anatomical characteristics (material properties, cross section size and shape, length) which in turn are influenced by animal (age, sex, size, breed, diet, and training history), and parameters associated with testing protocol (specimen history/test conditions that effects its material properties [wet/dry, temperature, time from death to removal to storage, time in storage], test loading [strain] rate, relative stiffness of the potting-fixtures-testing machine [K_pft_] to actual stiffness of the specimen [K_sa_], fixture constraints on the relative 3D motion between specimen ends, and load range of data used for stiffness calculation). Hanson et al. [1] considered all but the later three when discussing why stiffness values from all modes of testing (compression, torsion, bending) intact equine tibia by McDuffee et al. [15] were 2.5 to 6 times greater, and contributed this to McDuffee's greater loading rates. McDuffee et al. [2,15], Hanson et al. [1], and our compression testing was conducted in position control at constant ram velocity, with corresponding constant strain rate (ram velocity/exposed length of tibia or radius) = (0.58, 0.54), 0.00011, and 0.000018 (1/sec). McDuffee's compression strain rates were 527 and 491 times higher than Hanson's while ours' was 0.16 times lower. Similarly for torsion, (ram angular velocity/exposed length of tibia or radius) = (0.058, 0.054), 0.0017, and 0.00114 (deg/[mm*sec]); with McDuffee's being 34 and 32 times higher than Hanson's while ours' was 0.67 times lower. A quantitative comparison of strain rate for bending is not realistic due to load [2,15] versus position control [1] used, and that applied bending moment (strain) rate is not uniform over the length of a specimen for 3-point bending, ranging from 0 at the outer supports to a maximum at the central load application point.

Panjabi et al. [16] reported that cortical bone longitudinal modulus of elasticity increases with increase in strain rate by a factor of 1.5 for strain rate of 1500 compared to 1.0 (1/sec), thus explaining in part the greater stiffness observed by McDuffee et al. [2,15] compared to Hanson et al. [1]. If strain rate were the predominant factor, our length normalized measured stiffness values should be lower than or similar to those reported by Hanson et al. [1], since our strain rates were lower.

The order of magnitude difference in our K_3DN _results compared to those reported by Hanson et al. [1] is likely due to their K_pft _being relatively low compared to the K_sa _of the specimen. When using test machine ram displacement to calculate K_sm _of a specimen, ram displacement is the sum of that due to specimen deflection and PFT machine deflection, which includes bone crushing when applying bending loads directly to bone [1]. The theoretical model is two springs in series [17], one with stiffness K_sa _the other with K_pft_, resulting in a measured stiffness K_sm _= K_sa_/(1 + K_sa_/K_pft_). Ideally K_pft _is infinite (rigid PFT), thus K_sm _= K_sa_. The general rule-of-thumb when using ram displacement is to have K_pft _≥ 10*K_sa _so that K_sa _≥ K_sm _≥ (1/1.1) K_sa _and thus error is ≤ 9% when reporting K_sm _for K_sa_. Assuming K_3DN _= K_sa _and K_H _= K_sm _produces K_sa_/K_sm _ratios ranging from 7.5 to 19.6, which would require that Hanson's K_pft _= 0.15 K_sa _to 0.05 K_sa_. This is low but possible; however, Cowin stated that with many testing machines, the stiffness of the bone specimen is greater than that of the load frame [17]. Difference in K_pft _may also have contributed to the higher stiffness reported by McDuffee et al. [2,15] compared to Hanson et al. [1]. Use of direct measurement of test segment end motion to determine its stiffness is not subject to PFT stiffness error, which is one reason we selected to use 3D optical tracking and is reason for using an extensometer when testing for material modulus of elasticity [17]. The holes drilled in the radius to mount the LED rigid bodies were at the end cross sections of the test segment, thus having no effect on its stiffness.

Theoretically, in the absence of friction, the loading fixtures presented in this investigation allow 3D unconstrained components, of proximal relative to distal end motion of the radius, other than associated with the applied load component. Thus during loading, the ends of the specimen move into a relative 3D position that is dictated only by the 3D resistance of the (unsymmetrical or not) instrumented segment to the known (only) component of externally applied load such that mechanical equilibrium is achieved. The bending fixtures are the most likely to be subject to constraint by friction since the hardened cross bars were observed to indent the unhardened extension pipe surface during bending load to failure; the effect of this was reduced by periodically smoothing the pipe contact surfaces with a file.

Improvements could be made to reduce joint friction by using rollers incorporated into the 4-point bending load applicators [18]. Use of a commercially available universal joint has been reported [4] and was considered but not used since its pivot center would be several centimeters above or below the proximal or distal end of the bone being tested, respectively. Since the applied axial load remains coincident with the axis through the center of the universal joints, this would create, as load was increased, an ever increasing non-physiological lateral displacement of the plate from the axis of loading and thus bending moment on the specimen. Our goal was to keep the center of the spheres as close as possible to the ends of the radius being tested, so that the line of action of the axial applied load would remain close to the central point on each end of the radius. The authors' feel that the loading fixtures used and described in this manuscript, particularly axial and torsion, are a significant improvement towards achieving clinically relevant 3D unconstrained "worst case" in vivo loading simulation, compared to the fixtures reported in the literature [1,2,4].

Another advantage of this loading fixture design was that the same potting of the ends could be used to test a specimen in all three modes of loading: axial compression, torsion and bending. In addition the bending fixtures were capable of applying uniform 4-point bending moment in any transverse plane over the entire length of the instrumented specimen with no point of load application applied directly to bone or instrumentation hardware, and is independent of specimen and fixture cross section size and material since the center pivot assures equal forces at the inner application points. Thus the weakest aspect of an instrumented segment can be located, in contrast to it being predisposed to be at the location of the highest applied bending moment [1,2,15]. Also it avoids the problem of applying loads to non-circular bone cross sections and failure due to bone crushing at the point of transverse force application [1].

Stiffness is typically determined as the slope of the "linear portion" of each load versus displacement curve [1,2,15] leaving the range of data used being subjectively determined and variable, which can have a notable effect on the numerical value obtained. To reduce the subjectivity, we used data over a consistent load range to determine stiffness for each loading modality. The load range was selected so that all tests would have a linear load versus displacement characteristic within the range. Our load versus test machine ram displacement curves typically had lower slope during the first loading cycle compared to 2nd and 3rd loading cycles (the later two being similar), due to settling of bone-potting-fixture interconnections during the first cycle [17]. McDuffee et al. [2,15] and Hanson et al. [1] obtained stiffness from single cycle to failure tests, which could be a factor in the variability of and lower stiffness values observed.

The theoretical stiffness equations are based on the assumption that cross sections remain plane as load is applied, and thus corresponding experimental stiffness determination depends upon accurate 3D measurement of the position of these planes. For axial and torsional testing the initial position of the LEDs was pointing directly towards the 3 camera optical measuring head (Figures 2, 3) but were oriented at approximately 45 degrees for bending (Figures 4, 5). Accuracy in detecting the location of individual LEDs is known to deteriorate as their angle deviates from pointing directly at the measuring head [19]. This may explain why the average measured axial and torsional stiffness values were within the predicted range, while the average measured bending stiffness values were 0.97 to 1.5 times greater than the largest theoretically predicted stiffness for the four bending modes. This also might be an explanation for the occasional saw tooth type characteristic in some bending moment versus relative angular displacement graphs to which we used regression to fit a straight line to get the general trend and thus stiffness. Another possibility is that the single point of attachment to the radius of each rigid body did not produce an accurate representation of the 3D motion of the cross section plane. A recommended option would be to attach two rigid bodies to a C-ring with three pointed machine screws to make cortical bone contact at three points on the cross section plane. This would also eliminate the LED rigid body mounting hole's stress riser and potential for fracture initiation at this site, which did not occur in our tests to failure.

Long bone is known to be anisotropic and heterogeneous (differing in diaphyseal and epiphyseal properties), and is primarily elastic at low deformation rates [16,17]. The principal stress is longitudinal for compression and bending load modes. Published low strain rate longitudinal modulus of elasticity E determined by machine testing ranged from 17,000 to 22,600 (N/mm^2^) for human and bovine femur and tibia [17]. Torsional stiffness is a function of the longitudinal-circumferential shear modulus G, with published machine testing determined values ranging from 3300 to 5000 (N/mm^2^) for human and bovine femur and tibia [17]. Thus use of E = 18,000 and G = 4,615 for the short 50 mm long intact diaphyseal test segment produced representative theoretical stiffness values for use as a magnitude accuracy comparison reference.

## Conclusion

In conclusion, assuming negligible friction, the 3D loading-measurement system described in this manuscript: a) mimics unconstrained relative 3D motion between radius ends that occurs in clinical situations, b) applies uniform compression, torsion, and 4-point bending loads over the entire length of the test specimen, c) measures interfragmentary 3D relative motion between test segment ends to directly determine stiffness thus being void of PFT machine stiffness error, and d) has the resolution to detect differences in the 3D motion and stiffness of intact as well osteotomized-instrumented and ostectomized-instrumented equine radii. It is the authors' opinion that the 3D loading-measuring system described in this manuscript is capable of creating "worst case" clinically relevant loading conditions, and accordingly has the capacity to detect location and type of instrumented segment modes of failure and has the ability to produce more accurate stiffness results that are independent of PFT machine stiffness error.

## Appendix

Axial compression stiffness:   K_C _= AE/L   (N/mm)

Torsional stiffness:   K_T _= (1.745 * 10^-5^)*GK/L   (Nm/deg)

LM & ML bending stiffness:   K_LM _= (1.745 * 10^-5^)*EI_yy_/L   (Nm/deg)

CrCa & CaCr bending stiffness:   K_CC _= (1.745 * 10^-5^)*EI_xx_/L   (Nm/deg)

where:

D_p_, D_e _= Major diameter periosteal, endosteal respectively   (mm)

d_p_, d_e _= Minor diameter periosteal, endosteal respectively   (mm)

A = Cross section area   (mm^2^)

= (π/4) * (D_p_d_p _- D_e_d_e_)   for hollow ellipse

= (D_p_d_p _- D_e_d_e_)   for hollow rectangle

I_yy _= Area moment about y axis   (mm^4^)

= (π/64) * (D_p _^3^d_p _- D_e _^3^d_e_)   for hollow ellipse

= (1/12) * (D_p _^3^d_p _- D_e _^3^d_e_)   for hollow rectangle

I_xx _= Area moment about x axis   (mm^4^)

= (π/64) * (D_p_d_p _^3 ^- D_e_d_e _^3^)   for hollow ellipse

= (1/12) * (D_p_d_p _^3 ^- D_e_d_e _^3^)   for hollow rectangle

K = Polar moment type term   (mm^4^)

= (π/16)* [(D_p _^3^d_p_)(1-q^4^)]/[D_p _^2 ^+ d_p _^2^]   for hollow ellipse

= (1/12)* [(D_p_d_p_*(D_p _^2^+d_p _^2^)) - (D_e_d_e_*(D_e _^2^+d_e _^2^))]   for hollow rectangle

q = ratio of major endosteal/periosteal diameters = (D_e_/D_p_)

L = Length of the test segment = 50 mm

E = Cortical bone modulus of elasticity = 18,000   (N/mm^2^)

Range in literature (8,900 ≤ E ≤ 42,000)

G = Cortical bone shear modulus = 4,615   (N/mm^2^)

Range in literature (3,500 ≤ G ≤ 8,710)

## Abbreviations

3D: Three dimensional

ASIF: Association for the Study of Internal Fixation

CaCr: Caudal-to-cranial

CrCa: Cranial-to-caudal

K_3D_: Our intact measured stiffness results

K_3DN_: Our intact 50 mm long segment stiffness results normalized to Hanson et al test specimen length

K_H_: Hanson et al's ram displacement stiffness values

K_pft_: Potting-fixture-test machine stiffness

K_sa_: Actual stiffness of test specimen

K_sm_: Measured stiffness of test specimen

LM: Lateral-to-medial

LED: Light emitting diode

ML: Medial-to-lateral

PFT: Potting-fixture-test machine

PMMA: Polymethylmethacrylate

xyz_p_: Local orthogonal axes associated with proximal test segment end

xyz_d_: Local orthogonal axes associated with distal test segment end

## Competing interests

The author(s) declare that they have no competing interests.

## Authors' contributions

JCJ conceived the study, carried out the collection, potting, implanting, and testing of the specimens, and contributed to the experimental design. WLC designed the loading-measurement system and tested the specimens. DAW participated in the experimental design. All authors read and approved the final manuscript.
